# Identifying correlates and determinants of physical activity in youth: How can we advance the field?

**DOI:** 10.1016/j.ypmed.2016.02.040

**Published:** 2016-06

**Authors:** Andrew J. Atkin, Esther M.F. van Sluijs, James Dollman, Wendell C. Taylor, Rebecca M. Stanley

**Affiliations:** a MRC Epidemiology Unit & UKCRC Centre for Diet and Activity Research (CEDAR), University of Cambridge School of Clinical Medicine, Box 285, Institute of Metabolic Science, Cambridge Biomedical Campus, Cambridge CB2 0QQ, United Kingdom; b Alliance for Research in Exercise, Nutrition and Physical Activity, School of Health Sciences, University of South, Australia; c Department of Health Promotion and Behavioral Sciences, School of Public Health, The University of Texas Health Science Center at Houston, Houston, TX, USA; d Early Start Research Institute, Faculty of Social Sciences, University of Wollongong, Australia

**Keywords:** Children, Adolescents, Physical activity, Behaviour change, Correlates

## Abstract

This commentary provides a critical discussion of current research investigating the correlates and determinants of physical activity in young people, with specific focus on conceptual, theoretical and methodological issues. We draw on current child and adolescent literature and our own collective expertise to illustrate our discussion. We conclude with recommendations that will strengthen future research and help to advance the field.

## Highlights

• Steps to advance research on the correlates of physical activity are proposed.
• Precise use of terminology and more studies with a longitudinal design are needed.
• Identification of target behaviours and their context specific correlates is key.
• Innovation in the application and integration of behavioural theory is encouraged.
• Multivariable models should be used to identify independent associations.

## 1 Introduction

Physical inactivity is prevalent in the child and adolescent population (Hallal et al., 2012). The development and evaluation of interventions to promote physical activity in youth, therefore, is a public health priority, particularly in light of the limited effectiveness of interventions to date (Gillis et al., 2013, Metcalf et al., 2012). One explanation for the modest effect of existing interventions is that they have failed to adequately target the most important determinants of physical activity (Sallis and Owen, 1999). Whilst the volume of literature describing the correlates and determinants of physical activity in youth is relatively large, it is highly inconsistent in terms of findings and methodological quality (Craggs et al., 2011, Stanley et al., 2012, Uijtdewilligen et al., 2011). In order to develop more effective interventions, the quality of this evidence base requires improvement. The aim of this commentary is to critically discuss current conceptual and methodological practices that underpin research into the correlates and determinants of physical activity. Drawing upon the authors' collective expertise, we highlight key areas for consideration and provide recommendations to advance the field.

## 2 Study design and key terminology

At present the evidence base is dominated by cross-sectional studies, which do not allow for causal inferences to be made due to the risk of reverse causality. Examples of the use of terms such as ‘influences’ and ‘determinants’ in such studies abound in the literature. However, cross-sectional associations should be referred to as ‘correlates’ and the term ‘determinant’ reserved for exposures identified in longitudinal studies (Bauman et al., 2002). Although studies in which exposure assessment temporally precedes outcome assessment provide a stronger basis for drawing causal inferences, the possibility of bidirectional or reverse causal pathways should not be overlooked. The strongest evidence of causation arises from carefully controlled trials that manipulate a hypothesised causal factor and evaluate the impact on physical activity, in which case the term ‘mediator’ may be applied. We advocate more widespread use of longitudinal (observational or intervention) study designs and more accurate use of terminology to aid accurate interpretation. In the remainder of this commentary, we use the generic term ‘correlate’, unless referring specifically to longitudinal associations.

## 3 Behaviour and context specificity

Correlates studies too frequently utilise a highly aggregated outcome variable representing overall (weekly or habitual) physical activity. This approach masks the fact that each episode of physical activity consists of a specific set of behaviours, performed in a specific context. To maximise the predictive capacity of behavioural models, and comply with behavioural sciences theories, it is necessary to optimise the correspondence between exposure and outcome measures (Ajzen, 1991, Fishbein and Cappella, 2006, Giles-Corti et al., 2005, Institute of Medicine, 2002). Clarification of ‘behavioural context’ enhances specificity of behavioural models by highlighting key characteristics in terms of person, place, time, and activity type. These characteristics will differ across the broad range of activities children engage in, such as walking to school, participating on sports teams, playing active video games or attending after-school clubs, and the correlates of these behaviours, therefore, are likely to differ. The identification of target behaviours that contribute substantially to children's overall activity and their context-specific correlates should be a priority area for future research. One way of operationalising this approach is to consider the temporal characteristics of behaviour by focussing upon specific times of the day or week (Corder et al., 2013, Stanley et al., 2012).

## 4 Measurement issues

Until recently, the literature was dominated by the use of self- or proxy-reported measures of physical activity, which generally have poor validity (typical correlation with accelerometer assessed physical activity < 0.5) (Chinapaw et al., 2010). Physical activity measurement has improved through the use of objective monitoring (e.g. accelerometry) but studies applying these methods have generally identified fewer associations with candidate correlates and accounted for less variance in outcome variables than those that have relied upon self- or proxy-reports. This may reflect the influence of correlated errors in self-reports of exposures and outcomes, which serves to artificially inflate the proportion of variance that an exposure can account for in an outcome. Alternatively, it may be that participants' conceptualization of ‘physical activity’ differs from that which is assessed by an activity monitor. Where parents report their agreement with items regarding provision of support for their child's physical activity, for example, they may only be considering a subset of planned activities (such as attending sports club), whereas an activity monitor will capture the totality of children's activity. Such disparities are likely to attenuate associations in statistical models. This concern is particularly pertinent for young children, where parental reports are necessary because children are not capable of providing self-reports. Greater context specificity in exposure measurement will aid advancements in this area. Methodological developments to improve the assessment of specific activity behaviours will also be beneficial. Although the current generation of objective monitors enable the collection of time-stamped data on activity intensity, they are limited in their capacity to identify specific behaviours, which are still commonly captured through self-report. The use of multiple sensors in combination (e.g. accelerometry and GPS), advanced analytical methods (e.g. machine learning (Mannini and Sabatini, 2010)) or time-use methodologies (e.g. ecological momentary assessment (Foley et al., 2012, Stone and Shiffman, 1994)) that capture contextual information will help to overcome this limitation.

## 5 Theory and its application

Behavioural sciences theory can contribute to advancing understanding of the correlates of physical activity. Contemporary thinking advocates the application of an ecological framework to reflect the influence of, and interactions among, individual characteristics, social/cultural factors, the built environment, and policies (Sallis and Owen, 2002). Ecological models are particularly well suited for studying physical activity because they serve to highlight the plurality of potential influences on behaviour, as determined by the unique context in which behaviour occurs. They also provide a framework within which theoretical models that focus on a specific level of influence, such as the family, may be incorporated (Taylor et al., 1994). A key challenge that has received relatively little attention to date is to identify how constructs from different ecological levels interact (Dollman et al., 2007). This objective may require innovative thinking to identify how theories that operate at different ecological levels may be integrated or to identify potential pathways of influence that combine distal and proximal factors. More extensive use of behavioural sciences theory will facilitate the conduct of ‘hypothesis testing’ studies, in contrast to the ‘exploratory’ or ‘hypothesis generating’ approach more commonly seen in the epidemiological literature. As has been applied in the context of intervention research (Michie et al., 2011), the development of a common language and precise definitions of key constructs would also help to advance the field by facilitating data pooling and evidence synthesis.

## 6 Analytical approaches

Statistical models are used to examine the direction, magnitude and shape of the association between exposure and outcome variables and evaluate the likelihood that observed associations are due to chance. We suggest that regression models are preferable to simple hypothesis testing methods (e.g. ANOVA) because they provide a measure of the magnitude of an association. Multivariable models should be applied routinely, to enable identification of independent associations where multiple correlates are examined simultaneously and to control for confounding. As many hypothesised correlates of physical activity may themselves be correlated (e.g. parental physical activity and parental support for their child's activity), collinearity should be considered and appropriate analytical techniques (e.g. correlated component analysis) applied where necessary.

The correlates of physical activity may vary across population subgroups; therefore examining effect modification is valuable. However, some published papers proceed to conduct stratified analyses on the basis that physical activity levels differ between subgroups (e.g. boys are more active than girls). This represents a misunderstanding of the concept of moderation. Differing levels of physical activity does not necessitate that the association between an exposure and outcome will also vary between these groups. Moreover, there is a risk of overinterpreting between-group differences observed from stratified analyses, particularly when sample sizes differ between subgroups. Formal statistical tests should be conducted to examine interactions amongst correlates ‘within’ or ‘between’ levels of the ecological model and stratified analyses presented only where justified on this basis.

## 7 Conclusion and recommendations

A robust evidence base identifying the key correlates and determinants of physical activity is essential to inform the design of effective behaviour change interventions. Modest progress has been made in this regard, but further developments in theory, design and methodology are required to advance the field. In summary of the points raised in this commentary, we provide the following recommendations:

• Careful attention should be paid to the appropriate use of terminology.
• More studies that employ a prospective design, both observational and experimental, are required.
• Exposure and outcome assessment methods should be selected or developed where necessary to optimise context specificity.
• Behavioural sciences theories should be employed, singly or in combination, to inform study design and identify research questions.
• Multilevel behavioural sciences theories and frameworks are recommended to capture the context, timing, and complexity of children's physical activity.
• Analytical methods that provide an estimate of effect size and enable the control of confounding, in addition to evaluating the role of chance, should be applied routinely.

## Conflict of interest statement

The authors declare that there are no conflicts of interest.
